# Macromolecular prodrugs of ribavirin: towards a treatment for co-infection with HIV and HCV†
†Electronic supplementary information (ESI) available: Additional experimental details and figures. See DOI: 10.1039/c4sc02754j
‡A.A.A.S.; K.Z.; M.B.L.K.; B.W. contributed equally to this work.


**DOI:** 10.1039/c4sc02754j

**Published:** 2014-09-16

**Authors:** Anton A. A. Smith, Kaja Zuwala, Mille B. L. Kryger, Benjamin M. Wohl, Carlos Guerrero-Sanchez, Martin Tolstrup, Almar Postma, Alexander N. Zelikin

**Affiliations:** a Department of Chemistry Aarhus University , Aarhus C 8000 , Denmark . Email: zelikin@chem.au.dk; b Aarhus University Hospital , Aarhus C , Denmark; c iNANO Interdisciplinary Nanoscience Centre , Aarhus University , Aarhus C 8000 , Denmark; d CSIRO-Manufacturing Flagship , Clayton VIC , Australia; e Friedrich Schiller University , Jena , Germany

## Abstract

Human immunodeficiency virus (HIV) and hepatitis C virus (HCV) represent tremendous healthcare burdens with a large proportion of patients hosting the two viruses at the same time. An altered hepatic function and immunity as well as cross-interference of drugs make treatment of co-infection increasingly challenging. Herein we report the first design of macromolecular prodrugs (MP) with concurrent success in fighting HIV and alleviating hepatitis (liver inflammation). To achieve this, polymer compositions were systematically screened in a broad range of molar mass and content of ribavirin – a broad spectrum antiviral agent. For the first time, we report that ribavirin is efficacious in fighting HIV and in the form of MP, the treatment is safe, both in terms of lack of association of ribavirin with red blood cells and lack of toxicity upon cellular internalization. The lead polymer compositions were also potent in anti-inflammatory assays with relevance to viral hepatitis – thus making up formulations with potential for treatment of co-infection with HIV and HCV.

## Abstract 2

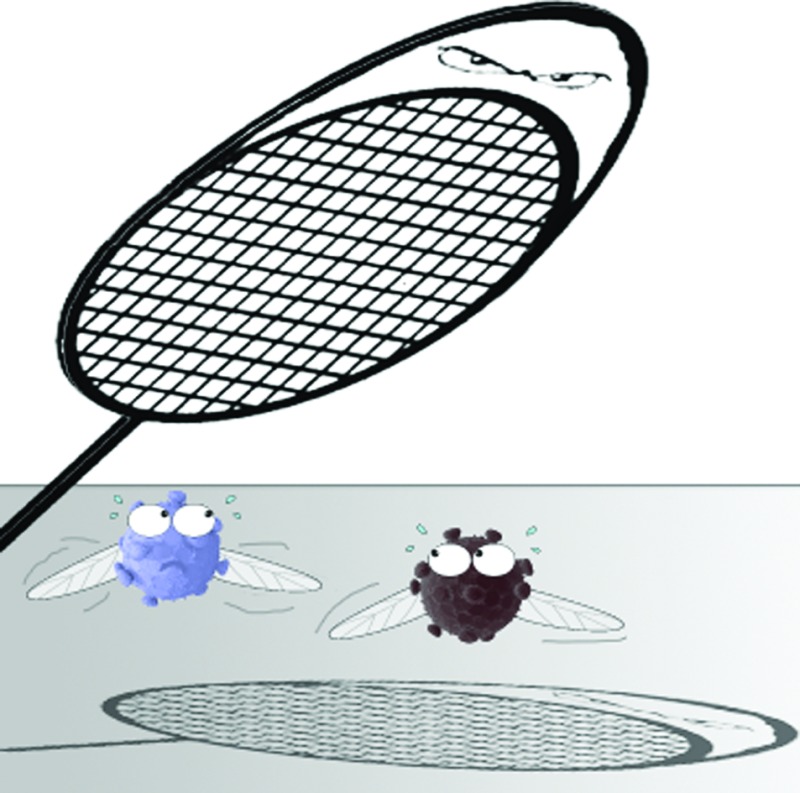
Macromolecular prodrugs of ribavirin were developed as blood safe formulations with capacity to fight inflammation and human immunodeficiency virus *in vitro*.

## 

Hepatitis C virus (HCV) and human immunodeficiency virus (HIV) together infect over 200 million people worldwide and are responsible for severe debilitating diseases such as viral hepatitis and AIDS. Keeping these viruses under control within the human body is a challenge for the global healthcare even when considering infections with either virus individually.^1^ A further burden is found in that there is a significant level of co-infection with these viruses – the United States Centre for Disease Control reports that one in four individuals positive for HIV is also positive for HCV.^2^ Altered hepatic function and compromised immunity of these patients, as well as the interference between the administered drugs,^3,4^ significantly complicate antiviral therapy. Development of therapeutic agents with activity against both HCV and HIV (or the diseases resulting therefrom) could therefore provide a solution to this global biomedical challenge. However, despite a significant degree of similarity between HCV and HIV replication and shared guidelines to the drug development,^1^ such dual-acting antiviral agents are yet to be identified.

The arsenal of antiviral therapeutic agents has solitary candidate drugs with a true broad spectrum of activity. Of these, ribavirin (RBV) is a powerful drug against influenza virus, Lassa fever virus *etc.* ^5^ and is the prime candidate in the anti-HCV treatment.^1^ For HCV in particular, more successful drugs such as sofosbuvir are currently entering the market, yet these too are administered together with RBV further highlighting the importance of this drug in antiviral treatments. In turn, activity of RBV against HIV is contested and is believed to be unreliable^6,3^ – at least not at the doses of RBV that are approved for clinical use. In part, this is due to the highly unfortunate and significant side effects of ribavirin such as accumulation in red blood cells leading to anemia^7,8^ – a severe dose-limiting phenomenon. We hypothesized that making a dual acting formulation against HCV and HIV is possible through optimization of delivery of RBV. In this work, we address this biomedical challenge using the tools of nanomedicine and specifically, through the synthesis of macromolecular prodrugs (MP).^9,10^

The field of polymer therapeutics in general,^11,12^ and that of MP of RBV, is characterized by a broad macromolecular parameter space. The nature of the polymer carrier, its average molar mass and drug loading may each play a decisive role in the overall success or failure of the formulation.^13^ In this work, we use a polymer with extensive characterization in biomedicine including advanced clinical trials, namely poly(*N*-(2-hydroxypropyl) methacrylamide), PHPMA.^11^ For an accelerated screen of the structure–function parameter space, we obtained a library of polymers with independently varied molar mass and content of RBV. Resulting MP were screened for activity in cell culture models with relevance to the viral hepatitis and anti-HIV research, in the latter case using the live infectious virus. In parallel, hemocompatibility of the polymers was evaluated through quantitative analysis of their association with red blood cells, hemolysis and agglutination. Using this methodology, we identified formulations of RBV that bypass the origin of the main side effect of this drug and have concurrent activity in HIV and HCV related assays. The specific novelty of this work lies in that, to our knowledge, we present the first example of such dual-acting macromolecular prodrugs. The lead prodrug compositions (molar mass and drug content) were blood-safe, proved to be equally active as the parent drug in preventing infectivity of HIV and were as efficacious as RBV in the inflammation read-out. We believe that these results will prove important in the development of antiviral prodrugs with broad spectrum of activity and specifically for the treatment of co-infections with HCV and HIV.

The synthesis of MP was accomplished *via* copolymerization of monomers corresponding to the carrier polymer and that with the functionality of the drug through a controlled radical polymerization technique, Reversible Addition Fragmentation chain Transfer (RAFT).^14,15^ The copolymerizations of HPMA with RBV methacrylate were performed on an automated synthesis platform with robotic handling of the solutions of monomers, chain transfer (RAFT) agent, and the initiator.^16^ The polymerizations were performed using dimethyl sulphoxide (DMSO) as a solvent to accommodate dissolution of ribavirin methacrylate to high monomer concentrations. Experiments were designed so as to synthesize four polymer series grouped by degree of polymerization (DP) and varying RBV methacrylate content within each series from 0 to 20 mol% (40 wt%). With exception of series with lowest targeted molar mass, polymer were obtained in duplicate of which one set of polymers was synthesized using monomer mixtures containing a methacrylate derivative of fluorescein (2 × equimolar to the RAFT agent). Fluorescent polymers are routinely used to monitor association of the MP with cells using fluorescence microscopy techniques and flow cytometry. Synthesis of two sets of polymers at similar conditions also allows to verify reproducibility of the syntheses with regards to the macromolecular characteristics of the polymers and their performance in drug delivery applications. Schematic illustrations of the chemical reactions and proton nuclear magnetic resonance (^1^H NMR) spectra of the starting materials and a resulting polymer are shown in Fig. 1A and B, respectively.

**Fig. 1:**
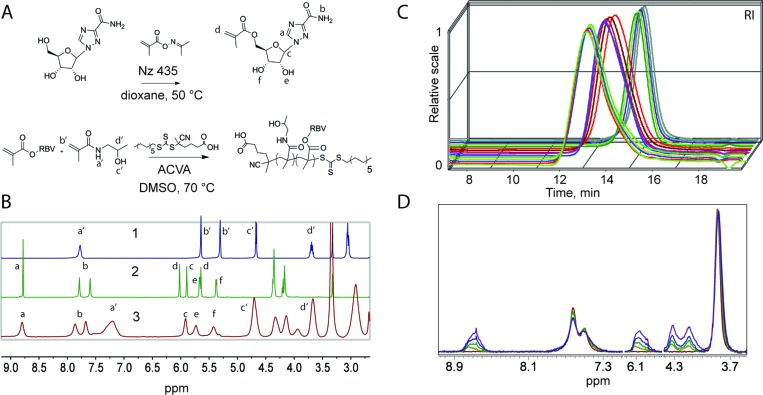
(A) Schematic illustration of the synthesis of RBV methacrylate and macromolecular prodrugs for RBV based on HPMA. (B) ^1^H NMR (*d*6-DMSO) spectra for HPMA (1), RBV methacrylate (2), and a representative MP (3) with the peaks at 5.4 and 5.7 ppm being the hydroxyls of RBV. (C) Size exclusion chromatography elution profiles for 15 representative polymers comprising polymer series with target degree of polymerization (left to right) 260, 150, and 50 (non-fluorescent series with [M]/[RAFT] 500, 250 and 50). (D): The superimposition of ^1^H NMR spectra (D_2_O) showing increasing RBV loading within one set of polymers with having a narrow mass range of 5–6 kDa with RBV loading increasing from 0% to 23 mol%. The signals stemming from RBV (3.9–4.4, 6.0–6.2, 8.5–8.9 ppm) are normalized to a signal from the PHPMA carrier (3.8 ppm).

The synthesized polymers were characterized per average molar mass and dispersity using an aqueous size exclusion chromatography (SEC) system equipped with an 8-angle light scattering detector (MALS), which allows for estimating absolute molecular weight of the polymers. SEC elution profiles corresponding to polymer series with the same target degree of polymerization revealed that the synthesized polymers indeed are characterized with near-matched elution profiles (Fig. 1C) speaking towards their uniform macromolecular characteristics. This finding is highly important to ensure that the sought after structure–function correlations are sound. For polymers within a series, NMR spectra revealed a gradually increasing content of RBV (Fig. 2D) illustrating the accomplished independent control over polymer DP and drug loading.

**Fig. 2:**
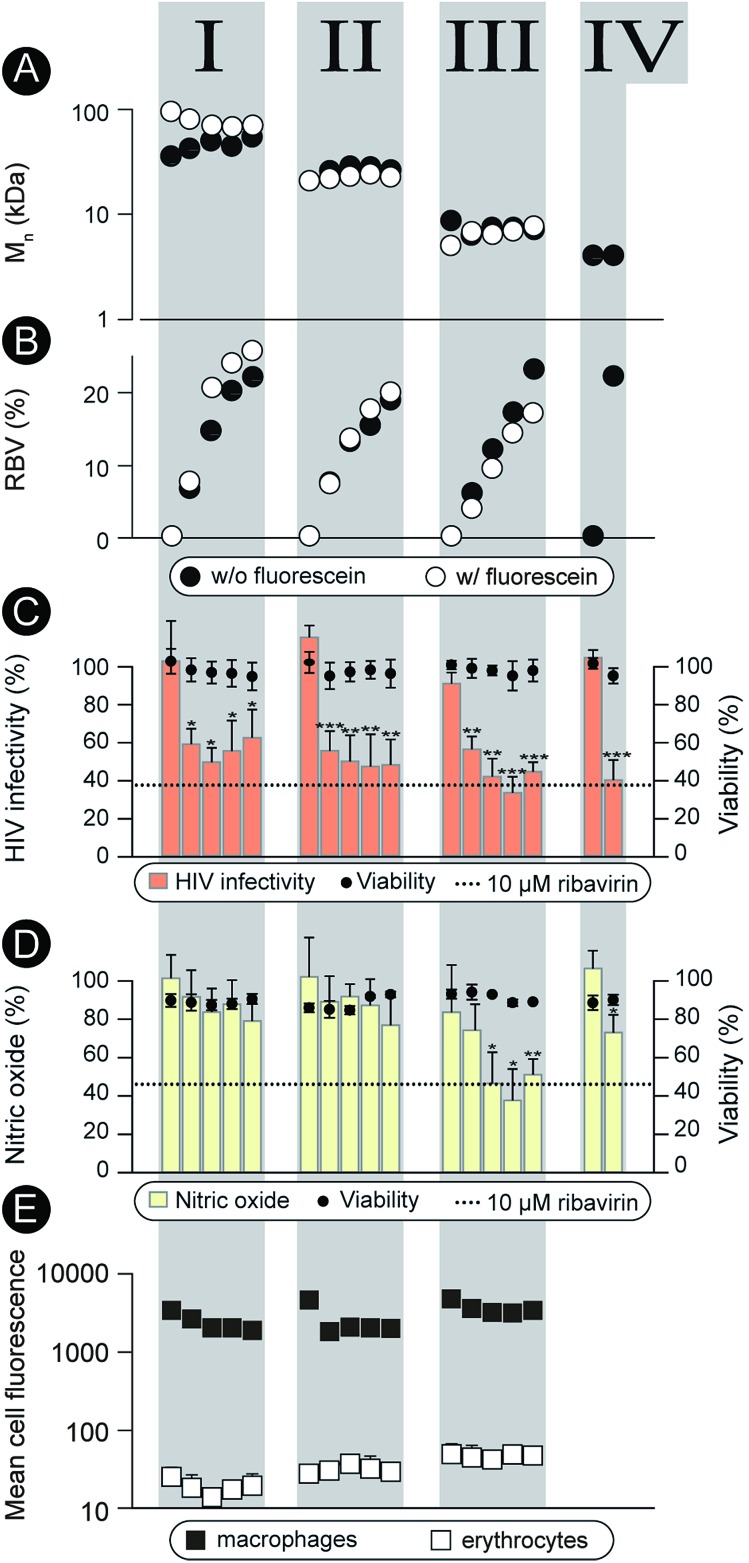
(A), (B): Graphic representation of *M*_n_ and content of RBV methacrylate in the polymer (mol%). Open and solid circles correspond to the polymer series synthesized with and without fluorescein methacrylate, respectively. (C) Activity of the non-labeled polymers in preventing infectivity of the HIV virus (TZM-bl cells, 0.1 g L^–1^, 24 h pre-incubation prior viral challenge, 48 h incubation until luciferase read-out). (D) Activity of the non-labeled polymers in the anti-inflammatory model (RAW macrophages, 0.1 g L^–1^ polymer, 24 h pre-incubation prior to LPS pro-inflammatory stimulation, 48 h until the final read-out); (E) flow cytometry data for association of the labeled polymers with macrophages and erythrocytes (0.1 g L^–1^ polymer concentration, 24 h incubation). Values of mean fluorescence were normalized to reflect variation in fluorescence of the polymers solutions. No results are given for series IV as no fluorescent analogs were prepared for this series due to its low molar mass. (C–E) Results are the average ± SD of at least three independent experiments (*n* = 3). Statistical significance is given in relation to the negative control. *P < 0.05, **P < 0.01, ***P < 0.001.

Characterization as discussed above was performed for all the synthesized polymers and their characteristics are graphically presented in Fig. 2A and B. The most important observation from this graph is that the syntheses afforded four well-defined polymer series grouped by similar molar mass and independently controlled content of RBV. For all the polymers, molar mass dispersity was at or below 1.3 and for most samples at or below 1.2 (see ESI†) thus revealing a good degree of control over the average molar mass of the polymers. The polymer series prepared in duplicate revealed closely matching values of the molar mass and the content of the drug on the polymer chains illustrating reproducibility of the syntheses. Altogether, Fig. 1 and 2 present the synthesis and characterization of macromolecular prodrugs of RBV with 17 unique compositions, *i.e.* molar mass and content of the drug (for numerical values of the number average molar mass (*M*_n_) and RBV content, see ESI†).

The synthesized polymers were characterized with regards to activity in the antiviral tests (non-fluorescent polymers) and tested for blood safety (fluorescent analogues with matched molar mass and RBV content). Against HIV, the polymers were employed to prevent infectivity of the replication-competent HIV-1 in TZM-bl cells using luciferase expression as a read-out^17^ (Fig. 2C). The pristine drug given at a clinically relevant level (10 μM RBV)^5^ inhibited viral infectivity but also exhibited a statistically significant cytotoxicity (cell viability 80 ± 10%, *p* < 0.01). It is worthy of note that while the magnitude of the cytotoxic effect is not overwhelming, HIV-infected patients face a life-long duration of treatment which aggravates even minor side-effects. For MP, each of the RBV-containing polymer samples revealed a pronounced antiviral effect, whereas pristine PHPMA had no effect on the viral infectivity. This result signifies efficient intracellular release of the drug from the carrier polymers. Moreover, MP were equally efficacious as RBV and at the same time were devoid of any noticeable cytotoxicity.

As part of the treatment against HCV and hepatitis, RBV does not exert a pronounced direct antiviral effect (at least not at the approved doses).^1^ It is proposed that activity of this drug relates to the inhibitory role of RBV on ionosine monophosphate dehydrogenase,^18^ specifically in liver-resident macrophages, through which it serves to counter-act the virus-induced inflammation.^19–21^ This activity can be quantified using the synthesis of nitric oxide (a marker for inflammation) as a read-out^22^ (Fig. 2D). This hepatitis-related read-out proved to be significantly more sensitive with regards to the structure–function relationship than the anti-HIV screen. Indeed, most of the prodrug samples were ineffective as carriers for RBV, likely revealing a molar mass – restricted cellular internalization of the polymer. However, of the 17 synthesized polymer compositions, at least 3 revealed an activity with efficacy matching that of RBV, Fig. 2, series III. Interestingly, these samples were all of molar mass around 8 kDa whereas polymers with greater or lower chain lengths were less effective. Identification of this molar mass “sweet spot” highlight the importance of the undertaken synthesis of a library of polymers with composition systematically changed within broad ranges of molar mass and drug content.

Fluorescently labeled polymer samples were used to monitor association of MP with macrophages or red blood cells through flow cytometry (Fig. 2E). In the latter case, the samples were used to concurrently quantify the hemolysis and agglutination, that is, polymer induced lysis (Fig. ESI 1†) and aggregation (Fig. ESI 2 and 3†) of the erythrocytes. Each of the polymers exhibited at least a 100-fold greater degree of association with macrophages than with the red blood cells thus successfully overcoming the origin of the main side effect of RBV, accumulation in the erythrocytes. Polymers caused no hemolysis or agglutination (Fig. ESI 1–3†) contributing to the safety profile of these prodrugs.

## Conclusions

The three independent screens revealed macromolecular prodrugs of RBV with negligible association with red blood cells and pronounced concurrent activity against HIV and inflammation, in the latter case with relevance to the viral hepatitis. At least 3 polymer compositions have efficacy matching that of RBV in both read outs. The identified molar mass “sweet spot” may reflect an optimal balance between efficiency of polymer uptake by the cells and deliverable payload per polymer chain. We have previously shown that lower molar mass may facilitate polymer cell entry in macrophages^13^ – a phenomenon evidently preserved for the HPMA-based MP. However, shortest polymer chains carry few (<5) copies of RBV and per event of cell entry, deliverable payload is much lower than for the higher molar mass chains with similar drug loading. With that, activity of MP to prevent infectivity of HIV-1 appears to be less restricted by the cell entry. This observation may be specific to the TZM-bl cells (a HeLa derived cell line) and may change upon testing of MP in T cells. Nevertheless, the novelty and importance of the above findings lie in that to our knowledge, we developed the first MP system with dual activity in both an HIV-antiretroviral and a hepatitis models. We anticipate that these data will be important for the development of safer treatment for the patients co-infected with HIV and HCV.

The experiments presented in this work were conducted *in vitro* and as such represent only the first but a very important step towards identifying the lead MP to be developed into a treatment against co-infection with HIV and HCV. The blood safety screen described above was done with human blood and these results are likely to be reflecting the *in vivo* settings. Also, anti-HIV work was done with live infectious virus and activity of the polymers is therefore tested against the real, not a model pathogen. Subsequent *in vivo* tests are warranted to reveal the suitability of these polymers with regards to pharmacokinetics and in particular biodistribution, and this will be the subject of our future work.

## Experimental section

Unless stated otherwise, all chemicals were purchased from Sigma-Aldrich and used without further purification. RBV was purchased from APIChem (China). Milli-Q water (resistivity 18.2 MΩ cm) was obtained from a Millipore Milli-Q Direct 8 system. Trypsin–EDTA 0.05%, fetal bovine serum (FBS, < 5 EU mL^–1^) and Presto Blue cell viability assay were purchased from Invitrogen. Cell Counting-Kit CCK-8 was purchased from Sigma-Aldrich. Britelite plus reagent for measuring activity of luciferase reporter system was purchased from Perkin-Elmer. RAW264.7 (murine monocyte macrophage cell line, ATCC) and TZM-bl cells (obtained from NIH AIDS Reagent Program, catalogue no. 8129) were routinely cultured in full culture medium containing Dulbecco's modified Eagle medium (DMEM), FBS (10%) and penicillin/streptomycin (1%) at 37 °C with 5% CO_2_.

### Monomer synthesis

RBV methacrylate was synthesized as previously reported.^23,13^

### Polymer synthesis

A Chemspeed Swing-SLT automated synthesizer was used for the parallel synthesis.^16^ The reactions were performed in a glass reactor block of 16 reactors (13 mL) with thermal jackets. These were connected in series to a Hüber thermoregulation system (–90 to 140 °C). The reactors were equipped with cold-finger reflux condensers and mixing was done by vortex agitation. The liquid transfers were performed by a 4-needle head, which was washed in between each transfer with DMSO. The DMSO reservoir was degassed by continuous nitrogen sparging. The needle head and tubing lines were also primed with DMSO prior to the transfers. The reaction vessels were heated to 135 °C and subjected to 10 cycles of vacuum and subsequent nitrogen filling prior to the experiments as to remove oxygen. The reaction mixtures were degassed through an automated parallel freeze–evacuate–thaw degassing method. All stock solutions were degassed by sparging with nitrogen for 15 min before being put in the Chemspeed. The desired aliquots of stock solutions and solvent from the reservoir were transferred to the reactors with the automated liquid handling system. Penultimately, the reactors were degassed through three automated freeze–evacuate–thaw cycles, before the reactors were heated to 70 °C while subjected to vortex agitation for the duration of the polymerization.

The polymerizations were performed with a monomer to RAFT ratio of 500, 250, 50, 35 and 20. These were done with a ribavirin methacrylate feed varying from 0–20 mol%. The following stock solutions in DMSO were prepared; HPMA 0.5 g mL^–1^, ribavirin methacrylate 0.38 g mL^–1^ 4-cyano-4-[(dodecylsulfanylthiocarbonyl)sulfanyl]pentanoic acid (RAFT agent) 50 mg mL^–1^ with 4,4′-azobis(4-cyanovaleric acid) (initiator) 8.7 mg mL^–1^ as a mixture. These stock solutions were aliquoted into the reaction vessels – see ESI† for full details. Fluorescent duplicates of the polymers with the monomer to RAFT ratios of 500, 250 and 50 were made by adding two equivalents of fluorescein methacrylate (with respect to RAFT agent) to the RAFT-initiator stock solution mixture.

### Polymer characterization

Size-exclusion chromatography (SEC) was performed using a system comprising a LC-20AD Shimadzu HPLC pump, a Shimadzu RID-10A refractive index detector and a DAWN HELEOS 8 light scattering detector along with a SPD-M20A PDA detector, equipped with a HEMA-Bio Linear column with 10 μm particles, a length of 300 mm and an internal diameter of 8 mm from MZ-Analysentechnik in series with a OHpak SB-803 HQ Shodex column with the dimensions 8.0 × 300 mm a particle size of 6 μm. The solvent used was water filtered through a 0.1 μm filter with 300 ppm sodium azide. The dn/dc used to calculate the molecular weights of the polymers were those of the carrier polymer (0.168 mL g^–1^, measured with MALS assuming full mass recovery). Proton nuclear magnetic resonance (^1^H NMR) spectra were obtained with a Varian Mercury 400 MHz NMR spectrometer. Ribavirin content of the polymers was determined from ^1^H NMR by comparing intensities of the signals of HPMA to RBV, *i.e.* the signal at 3.7 ppm (exclusively from HPMA in D_2_O) and the signal at 6.15, which stems from RBV. For numerical values of the *M*_n_ and RBV, mol% see ESI.†

### Anti-HIV activity

Anti-HIV activity of RBV bearing polymers was conducted using TZM-bl cell line as reported earlier.^17^ This cell line is engineered with a luciferase/β-galactosidase reporter system under control of an HIV long terminal repeat promoter, and expresses the CD4 receptor and CCR5/CXCR4 co-receptors which are indispensable for HIV to infect the cell. Briefly, TZM-bl cells were preincubated with polymers at conc. 0.1 g L^–1^ for 24 h, following which cells were infected with HIV-1 Bal strain (14 TCID50). After 48 h level of infection was quantified *via* luciferase assay (Perkin-Elmer). Cell viability was measured using Cell Counting-Kit (Sigma-Aldrich).

### Anti-inflammatory assay

Intracellular activity of RBV bearing polymer conjugates was assayed based on the anti-inflammatory activity of RBV.^22^ In short, polymers were incubated at the indicated concentration with a macrophage cell line (RAW264.7) and subsequently stimulated with lipopolysaccharide. Relative nitric oxide (NO) levels and viability were read-out through a multiplexed Griess^24^ and commercial metabolic activity (PrestoBlue) assay.

### Haemocompatibility

Haemocompatibility of RBV conjugated polymers were assessed as described earlier.^23,25^ In short, human red blood cells (RBC) were obtained from Skejby Hospital blood bank, cryopreserved in 54.7 mM glycerol, 0.050 M NaPO_3_, 0.37 mM NaCl, and on the day of experiment washed in PBS (3 × 10 mL, 5 min, 400 rpm). In a 96-well round-bottomed multiplate, RBCs (45 μL) were subjected to polymers (5 μL) and incubated 24 h (37 °C, 300 rpm). A blank (untreated cells), negative control (PBS) and positive control (50% Milli-Q water) were included. After incubation, the cells were washed with phosphate buffered saline (PBS) (150 μL, centrifugation 5 min, 400 rpm) and supernatant (50 μL) was analysed for haemolysis (UV-Vis absorbance, 541 nm). The remaining cell suspension was diluted 1 : 1000 with PBS for analysis through flow cytometry, with 10 000 events analysed per sample. Haemolysis is presented relative to the positive controls and a negative blank (untreated cells) and was calculated using respective values of optical density (A) as 100% × (A_sample_ – A_blank_)/(A_positive control_ – A_blank_). Visualization of RBCs was performed using an inverted microscope (Axio Observer Z1, Zeiss, Germany).

### Data analysis

Microsoft Excel 2010 was used to analyze results from cell experiments and statistical significance was validated through a two-tailed heteroscedastic *t*-Test, with results considered statistically relevant if *P* < 0.05 (*), *P* < 0.01 (**) and *P* < 0.001 (***).
