# Supplementary material for: Macromolecular prodrugs of ribavirin: towards a treatment for co-infection with HIV and HCV
Source: Chem Sci. 2014 Sep 16;6(1):264–9. doi: 10.1039/c4sc02754j (PMC5435870; doi:10.1039/c4sc02754j)
Supplement: Supplementary file 1 [file SC-006-C4SC02754J-s001.pdf]

Electronic Supplementary Material (ESI) for Chemical Science.
This journal is © The Royal Society of Chemistry 2014

Supporting Information

Macromolecular prodrugs of ribavirin: Towards a treatment for co-infection with HIV and HCV.

Anton A.A. Smith,[1,#] Kaja Zuwala,[1,2,#] Mille B. L. Kryger,[1,3,#] Benjamin M. Wohl,[1,3,#] Carlos Guerrero-Sanchez,[4,5] Martin Tolstrup,[2] Almar Postma,[4] Alexander N. Zelikin[1,3,*]

[1] Department of Chemistry Aarhus University, Aarhus C 8000, Denmark
E-mail: zelikin@chem.au.dk
[2] Aarhus University Hospital, Aarhus C, Denmark
[3] iNANO Interdisciplinary Nanoscience Centre, Aarhus University, Aarhus C 8000, Denmark
[4] CSIRO - Manufacturing Flagship, Clayton VIC, Australia
[5] Friedrich Schiller University, Jena, Germany

Polymer synthesis. A Chemspeed Swing-SLT automated synthesizer was used for the parallel synthesis.[16] The reactions were performed in a glass reactor block of 16 reactors (13 mL) with thermal jackets. These were connected in series to a Hüber thermoregulation system (−90 °C to 140 °C). The reactors were equipped with cold-finger reflux condensers and mixing was done by vortex agitation. The liquid transfers were performed by a 4-needle head, which was washed in between each transfer with dimethyl sulfoxide (DMSO). The DMSO reservoir was degassed by continuous nitrogen sparging. The needle head and tubing lines were also primed with DMSO prior to the transfers. The reaction vessels were heated to 135 °C and subjected to 10 cycles of vacuum and subsequent nitrogen filling prior to the experiments as to remove oxygen. The reaction mixtures were degassed through an automated parallel freeze-evacuate-thaw degassing method. All stock solutions were degassed by sparging with nitrogen for 15 min before being put in the Chemspeed. The desired aliquots of stock solutions and solvent from the reservoir were transferred to the reactors with the automated liquid handling system. Penultimately, the reactors were degassed through three automated freeze-evacuate-thaw cycles, before the reactors were heated to 70 °C while subjected to vortex agitation for the duration of the polymerization.

The polymerizations were performed with a monomer to RAFT ratio of 500, 250, 50, 35 and 20. These were done with a ribavirin methacrylate feed varying from 0-20 mol%. The following stock solutions in DMSO were prepared; HPMA 0.5 g/mL, ribavirin methacrylate 0.38 g/mL, 4-cyano-4-[(dodecylsulfanylthiocarbonyl)sulfanyl]pentanoic acid 50 mg/mL with 4,4'-azobis(4-cyanovaleric acid) 8.7 mg/mL as a mixture. Fluorescent duplicates of the polymers with the monomer to RAFT ratios of 500, 250 and 50 were made by adding two equivalents of fluorescein methacrylate (with respect to RAFT agent) to the RAFT-initiator stock solution mixture.

These stock solutions were aliquoted into the reaction vessels according to the following table :

Polymer synthesis

The table below details the reagent compositions for the polymerizations with monomer to RAFT ratios of 500, 250 and 50, these polymerizations were run at 70 °C for 12 hours.

	HPMA (mL)	RBV-MA (mL)	Initiator + RAFT (mL)	Solvent (mL)
Reactor 1	0.900	0.000	0.051	1.049
Reactor 2	0.856	0.129	0.051	0.964
Reactor 3	0.810	0.258	0.051	0.881
Reactor 4	0.765	0.387	0.051	0.797
Reactor 5	0.720	0.517	0.051	0.712
Reactor 6	0.900	0.000	0.102	0.998
Reactor 7	0.856	0.129	0.102	0.914
Reactor 8	0.810	0.258	0.102	0.831
Reactor 9	0.765	0.387	0.102	0.746
Reactor 10	0.720	0.517	0.102	0.661
Reactor 11	0.900	0.000	0.510	0.592
Reactor 12	0.856	0.129	0.510	0.507
Reactor 13	0.810	0.258	0.510	0.425
Reactor 14	0.765	0.387	0.510	0.340
Reactor 15	0.720	0.517	0.510	0.255

This scheme corresponds to the following amounts being added to each reactor:

	HPMA (mmol)	RBV-Methacrylate (mmol)	RAFT (μmol)	Initiator (μmol)	Fluorescein methacrylate (μmol)
Reactor 1	3.14	0.00	6.29	1.57	12.58
Reactor 2	2.99	0.16	6.29	1.57	12.58
Reactor 3	2.83	0.31	6.28	1.57	12.58
Reactor 4	2.67	0.47	6.29	1.57	12.58
Reactor 5	2.52	0.63	6.29	1.57	12.58
Reactor 6	3.14	0.00	12.58	3.15	25.15
Reactor 7	2.99	0.16	12.58	3.15	25.15
Reactor 8	2.83	0.31	12.58	3.15	25.15
Reactor 9	2.67	0.47	12.58	3.15	25.15
Reactor 10	2.52	0.63	12.58	3.15	25.15
Reactor 11	3.14	0.00	62.90	15.73	125.78
Reactor 12	2.99	0.16	62.90	15.73	125.78
Reactor 13	2.83	0.31	62.90	15.73	125.78
Reactor 14	2.67	0.47	62.90	15.73	125.78
Reactor 15	2.52	0.63	62.90	15.73	125.78

The same procedure is done for the reactions with the monomer to RAFT ratios of 35 and 20, these polymerizations were run at 70 °C for 24 hours.

	HPMA (mL)	RBV-MA (mL)	Initiator + RAFT (mL)	Solvent (mL)
Reactor 1	0.675	0.000	0.389	0.736
Reactor 2	0.642	0.097	0.389	0.673
Reactor 3	0.607	0.194	0.389	0.611
Reactor 4	0.574	0.291	0.389	0.547
Reactor 5	0.540	0.388	0.389	0.483
Reactor 6	0.675	0.000	0.680	0.445
Reactor 7	0.542	0.388	0.680	0.192

Corresponding to the following amounts:

	HPMA (mmol)	RBV-Methacrylate (mmol)	RAFT (µmol)	Initiator (µmol)
Reactor 1	2.36	0.00	67.40	16.84
Reactor 2	2.24	0.12	67.40	16.84
Reactor 3	2.12	0.24	67.40	16.84
Reactor 4	2.00	0.35	67.40	16.84
Reactor 5	1.89	0.47	67.40	16.84
Reactor 6	2.36	0.00	117.90	29.48
Reactor 7	1.89	0.47	117.90	29.48

Polymer characterization

The analysis of the polymerizations (SEC and ^{1}H NMR) revealed the following characteristics ***(Polymer highlighted in bold were used in further cell experiments, see Main text)***

The non-fluorescent polymers with M:RAFT 500-50

Series	RBV feed	RBV content	M:RAFT	Conversion	Mn (kDa, calc)	Mn (kDa, GPC)	*Đ*	DP (GPC)
**I-1**	**0%**	**0%**	**500**	**49%**	**35.3**	**34.6**	**1.28**	**242**
**I-2**	**5%**	**7%**	**500**	**48%**	**37.8**	**40.6**	**1.23**	**261**
**I-3**	**10%**	**15%**	**500**	**55%**	**46.8**	**48.6**	**1.19**	**284**
**I-4**	**15%**	**20%**	**500**	**52%**	**46.8**	**42.8**	**1.28**	**236**
**I-5**	**20%**	**22%**	**500**	**52%**	**47.0**	**52.9**	**1.21**	**286**
**II-1**	**0%**	**0%**	**250**	**54%**	**19.6**	**20.0**	**1.26**	**140**
**II-2**	**5%**	**7%**	**250**	**62%**	**24.4**	**24.9**	**1.18**	**158**
**II-3**	**10%**	**13%**	**250**	**62%**	**26.0**	**27.8**	**1.15**	**165**
**II-4**	**15%**	**15%**	**250**	**61%**	**26.3**	**27.2**	**1.15**	**158**
**II-5**	**20%**	**19%**	**250**	**66%**	**29.1**	**25.5**	**1.13**	**142**
	0%	0%	50	80%	6.1	6.7	1.16	47
	5%	7%	50	82%	6.7	7.6	1.12	49
	10%	12%	50	79%	6.9	7.8	1.10	47
	15%	16%	50	81%	7.3	8.4	1.07	48
	20%	22%	50	81%	7.7	8.8	1.06	47

The non-fluorescent polymers with M:RAFT 35-20

Reaction	RBV feed	RBV content	M:RAFT	Conversion	Mn (kDa, calc)	Mn (kDa, GPC)	*Đ*	DP
**III-1**	**0**	**0**	**35**	**99%**	**5.0**	**8.1**	**1.20**	**57**
**III-2**	**5%**	**6%**	**35**	**99%**	**5.3**	**6.1**	**1.12**	**39**
**III-3**	**10%**	**12%**	**35**	**99%**	**5.6**	**7**	**1.06**	**42**
**III-4**	**15%**	**17%**	**35**	**99%**	**5.8**	**6.9**	**1.07**	**39**
**III-5**	**20%**	**23%**	**35**	**99%**	**6.0**	**6.4**	**1.09**	**34**
**IV-1**	**0%**	**0%**	**20**	**99%**	**2.8**	**3.7**	**1.12**	**26**
**IV-2**	**20%**	**22%**	**20**	**99%**	**3.3**	**4.1**	**1.03**	**22**

The fluorescent polymers with M:RAFT 500-50

Reaction	RBV feed	RBV content	M:RAFT	Conversion	Mn (kDa, calc)	Mn (kDa, GPC)	*Đ*	DP
**I-1**	**0%**	**0%**	**500**	**24%**	**17.4**	**92.1**	**1.24**	**643**
**I-2**	**5%**	**8%**	**500**	**24%**	**18.9**	**77.7**	**1.14**	**498**
**I-3**	**10%**	**20%**	**500**	**29%**	**25.9**	**67.8**	**1.21**	**381**
**I-4**	**15%**	**24%**	**500**	**38%**	**35.1**	**65.7**	**1.18**	**358**
**I-5**	**20%**	**25%**	**500**	**53%**	**49.7**	**67.7**	**1.19**	**363**
**II-1**	**0%**	**0%**	**250**	**50%**	**17.9**	**20.0**	**1.29**	**140**
**II-2**	**5%**	**7%**	**250**	**60%**	**23.3**	**21.1**	**1.28**	**136**
**II-3**	**10%**	**13%**	**250**	**64%**	**26.5**	**22.2**	**1.25**	**134**
**II-4**	**15%**	**17%**	**250**	**66%**	**28.3**	**23.4**	**1.21**	**135**
**II-5**	**20%**	**20%**	**250**	**64%**	**28.3**	**22.0**	**1.13**	**124**
**III-1**	**0%**	**0%**	**50**	**80%**	**5.7**	**4.9**	**1.31**	**34**
**III-2**	**5%**	**4%**	**50**	**82%**	**6.1**	**6.4**	**1.19**	**43**
**III-3**	**10%**	**9%**	**50**	**83%**	**6.6**	**6.1**	**1.21**	**38**
**III-4**	**15%**	**14%**	**50**	**81%**	**6.8**	**6.5**	**1.14**	**39**
**III-5**	**20%**	**17%**	**50**	**80%**	**6.8**	**6.9**	**1.07**	**40**

Additional graphs

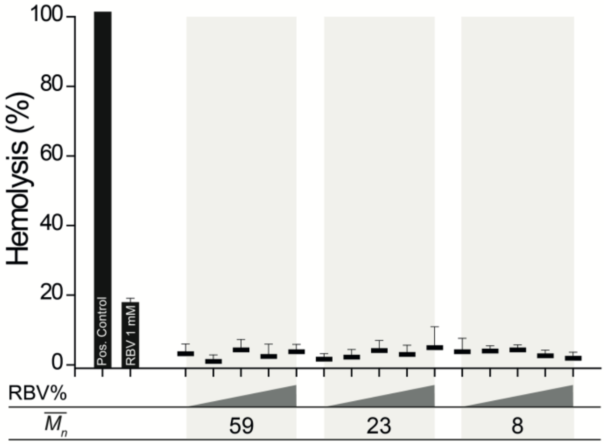

Figure SI 1. Haemolysis of erythrocytes upon incubation with macromolecular prodrugs at 100 μg/mL concentration over 24 h at 37 °C. Shown results are the average ± SD of three independent experiments (n=3). Depicted RBV content is for illustrative purposes only, for actual numerical values see the tables above.

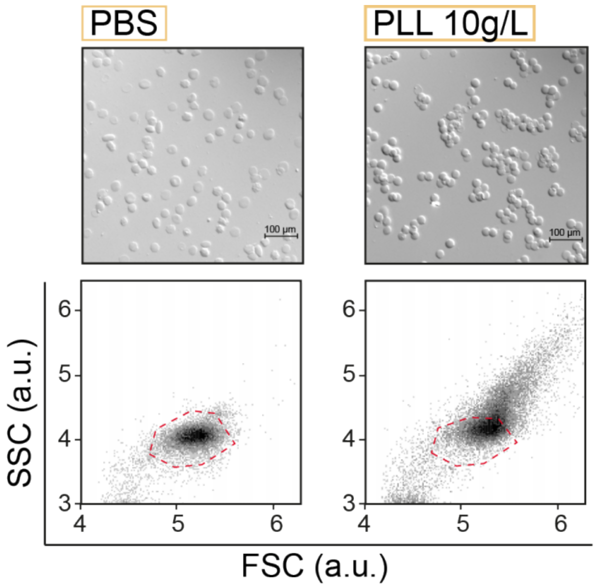

Figure SI 2. Top panel, microscope pictures illustrating PBS treated erythrocytes and PLL treated cells, the latter introducing aggregation of RBCs. Bottom panel, dot-plots from flow cytometry analysis of erythrocytes incubated with PBS and PLL (10 g/L) during 24 h at 37 °C.

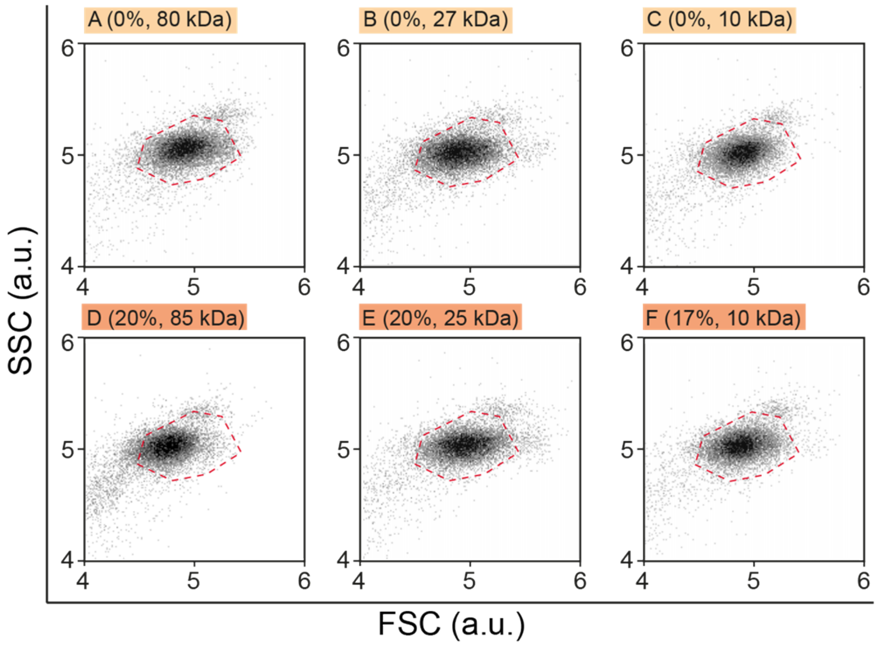

Figure SI 3. Dot-plots from flow cytometry analysis of erythrocytes incubated with MPs at 100 µg/mL during 24 h at 37 °C, with erythrocytes treated with PHPMA of similar M_n, with and without RBV: Top three panels showing 0%, 92 kDA, 0%, 20 kDa, and 0%, 5 kDa. Bottom panels showing 20%, 68 kDa, 20%, 22 kDa, and 17%, 7 kDa.
